# Quasi-Static and Fatigue Properties of Thermoset Sandwiches with 3D Continuous Fibre Reinforced Polyurethane Foam Core

**DOI:** 10.3390/ma15030764

**Published:** 2022-01-20

**Authors:** Kay Schäfer, Daisy Nestler, Lothar Kroll

**Affiliations:** 1 Endowed Chair Textile Plastic Composites and Hybrid Compounds, Faculty of Mechanical Engineering, Technical University of Chemnitz, 09126 Chemnitz, Germany; Daisy.Nestler@mb.tu-chemnitz.de; 2 Department of Lightweight Structures and Polymer Technology, Faculty of Mechanical Engineering, Technical University of Chemnitz, 09126 Chemnitz, Germany; Lothar.Kroll@mb.tu-chemnitz.de

**Keywords:** sandwich design, polyurethane foam, spacer fabric, thermoset composite, reaction injection moulding, resin transfer moulding, mechanical properties, fatigue, floor assembly, snow groomer

## Abstract

Innovative materials for substituting metals are required to reduce the mass of moving components. This decreases the CO_2_ emissions of overall systems. A thermoset sandwich for high mechanical properties and thermal insulation is presented in this paper. It has an innovative 3D continuous reinforced core, which allows the optimisation of the substance exploitation by wide-ranging possibilities of fibre orientation. This was demonstrated with three sandwich variants. The reference had no core reinforcement and the other two were reinforced with different spacer fabrics. The process chain for the manufacturing consists of Structural Reaction Injection Moulding (SRIM) and Vacuum Assisted Resin Transfer Moulding (VARTM). Significant increases in absolute as well as specific characteristic values were demonstrated by the reinforcement in a compression and bending test. It was also shown that quasi-static characteristic values under fatigue loading are maintained to a greater extent with the core reinforcement. The sandwich material was applied as a floor assembly for a snow groomer. The design was tailor-made for the mechanical, thermal and acoustic requirements. This proved the transferability of the process chain for manufacturing samples to the production of large-volume components with complex geometry.

## 1. Introduction and State of the Art

Forces can be directed through components according to the load requirements with fibre-reinforced plastics (FRP). Mass reductions of 30% compared to aluminium and 50% compared to steel can be achieved with FRP as substitution material for this reason [1]. The fuel combustion and consequently the CO_2_ emission of overall systems can be reduced as a result. Automotive engineering gives an example of the general relationship. Decreasing the total mass of a vehicle by 10% leads to a fuel saving of 4–6% [2]. Using FRP in a sandwich design with a foamed core creates additional lightweight potential. The resulting quasi-static mechanical properties are generally much higher than the sum of the materials used for the layers [3]. Further mass is saved through the porosity of the core [4]. This also provides excellent crashworthiness and heat insulating properties [5,6]. There is a large variety of plastics and fibres on the market. Different requirement profiles can be fulfilled by a targeted selection. The advantages and disadvantages of the production processes of the different combinations have to be taken into account thereby. The most important aspect is the distinction between thermosets and thermoplastics.

Thermoset sandwiches are manufactured from low-viscosity chemicals. Nearly any component shape can be formed, therefore. Reinforcing structures can also be impregnated without additional effort [7]. Thermosets have disadvantages after the lifetime of the component because no circular recycling is possible [8]. They are industrially only energetically utilized or disposed, which in each case burdens the environment [9]. At least producing from renewable raw materials is possible [10,11]. This prevents an additional greenhouse effect. Mostafa et al. presented the typical process for the manufacturing of thermoset sandwiches [12]. A polyurethane (PUR) foam core is first produced in the reaction injection Moulding (RIM). The facings are subsequently manufactured on the core in hand lamination, vacuum infusion or resin transfer moulding (RTM).

Thermoplastic sandwiches are produced from a high-viscosity melt which shows shrinkage during cooling. This restricts the freedom in component design and makes it difficult to fully impregnate textiles for reinforcement [7]. The great advantage of thermoplastics is their recyclability in a circular economy [8]. Menrath et al. showed the process chain for the production of thermoplastic sandwiches [13]. Continuous fibre-reinforced facings are manufactured by pressing. Two of them were inserted into an injection mould. A gas-loaded thermoplastic melt is introduced in between, forming the core and bonding the facings. The sandwich can be demoulded after the structure has cooled down.

The combination of thermosets and thermoplastics to manufacture sandwiches is also state-of-the-art. This enables the targeted use of the above-mentioned advantages of thermosets and allows partial recycling after the lifetime of the component by separating the layers. Avila et al. developed sandwiches with thermoset facings and thermoplastic core and the authors of this paper investigated the opposite combination [14,15].

The work described in this paper advances the state of the art with a completely thermoset sandwich with 3D continuous fibre reinforced core. This offers together with fibre-reinforced facings a maximum of possibilities for fibre orientation and consequently for substance exploitation. Great lightweight potential results in large-volume components with complex geometry where a long service life is planned. The technological prerequisites for this are basically available. The fibre reinforcement of compact thin thermosets is obviously state-of-the-art [16]. A current research topic is the 3D continuous fibre reinforcement of foam cores in contrast. This has not yet found an industrial application. Only the processing of short and long fibres in Reinforced Reaction Injection Moulding (RRIM) and 2D textiles in Structural Reaction Injection Moulding (SRIM) is established [17,18,19]. Spacer fabrics consisting of two fabric surfaces which are kept at a defined distance by spacer threads [20]. The suitability in principle of these structures for the reinforcement of PUR foams has been investigated and was also applied in this work [21,22,23,24].

The mechanical principle of sandwiches is very well described in the literature [3]. The most important load is bending. The facings are subjected to compressive and tensile forces thereby. The sandwich core and the component transition areas are stressed by shear forces. Experimental bending tests on sandwiches with PUR foam core have often been carried out [25]. The stress is thereby linear to the strain until failure occurs. This is expressed by a considerable drop in the measurement curve [12]. Typical failures caused by the described forces are the delamination of the facings and a diagonal fracture through the core [26]. The facings can break at the load application point as well [27]. The characteristics of the sandwich core under compressive loading are also important for keeping the facings at distance. This has also already been investigated for PUR foams. The deformation behaviour can be divided into three stages [28]. The beginning is characterised by elastic bending of the cell walls vertically to the load direction. The stress in the foam is linear to the compression thereby. The cell layers collapse one after another in the second stage. Reversible buckling of the cell walls occurs in flexible foams [29]. Irreversibly plastic is this process for rigid foams. The cell walls also rupture in this case [30]. The stress in the foam is always constant in relation to the compression during these deformation processes. Densification characterises the third stage, which leads to a progressive rise in stress with increasing compression.

Fatigue performance is also of great importance in most areas of sandwich application. Foam cores have to be investigated especially regarding this because they are usually hidden between the facings for decades and receive there repeating stresses [31]. Resulting structural damage remains unnoticed as a result. Delamination of the facings, crack formation and propagation as well as finally disintegration can occur [32]. This only becomes visible through total failure of the sandwich or after the lifetime of the component when it is disassembled. The following investigations deal with this topic.

Mathieson et al. investigated a sandwich consisting of glass-fibre reinforced thermoset facings and a PUR rigid foam core quasistatically and dynamically in a bending test [26]. Fatigue strength of 45% of the quasi-static strength was demonstrated for at least 2 × 10^6^ cycles at 0.5 Hz. Diagonal fracture through the core occurred consistently at higher loads. Sharma et al. proved the dependence of frequency on fatigue for a comparable sandwich design and also in a bending test [33]. Samples were loaded to 65% of their quasi-static strength for 10^6^ cycles. The sandwiches failed at 1 Hz just after 7.5 × 10^5^ cycles and at 5 Hz already after 1.5 × 10^3^ cycles. Sharaf et al. investigated the influence of the weight per volume of the foam core on the bending properties of a similar sandwich design [27]. The quasi-static bending strength increased by 2.5 times through the increase from 31 kg/m^3^ to 63 kg/m^3^. The four load levels 33%, 60%, 85% and 95% of the quasi-static strength were applied to each sample for four cycles in a dynamic test. Failure only occurred with the higher weight per volume through a diagonal fracture in the core. Greater energy absorption was measured for the lower weight per volume. Demirel et al. investigated the fatigue of PUR flexible foam under compressive loading. The weight per volume was varied between 14 kg/m^3^ and 32 kg/m^3^ using six variants [34]. A fatigue stress of 1.2 MPa and a frequency of 1.25 Hz were used. The quasi-static compressive stress values were recorded after 8 × 10^3^ and 8 × 10^4^ cycles. These were finally compared. The compressive stress value at 25% deformation decreased by 20% to 31% and at 65% deformation by 11% to 57%. The loss was lower with increasing weight per volume.

No experimental studies on the influence of fibre reinforcement of plastic foam cores on the fatigue performance of sandwiches are known. That is addressed in this paper. The quasi-static and fatigue compressive and bending properties of the developed thermoset sandwich with 3D continuous fibre reinforced rigid PUR foam core were investigated.

The application of plastic sandwiches with a foam core for components with fatigue performance requirements has already been demonstrated. Examples are wall cladding panels, bridges, ships and wind turbine rotor blades [27,31,35,36]. This paper presents the application of the investigated sandwich in special vehicle construction. A floor assembly for a snow groomer was developed and manufactured. The reinforcing effect of spacer fabrics was also demonstrated for the component by a mechanical test.

## 2. Experimental Work

### 2.1. Sandwich Design and Materials

**Figure 1:**
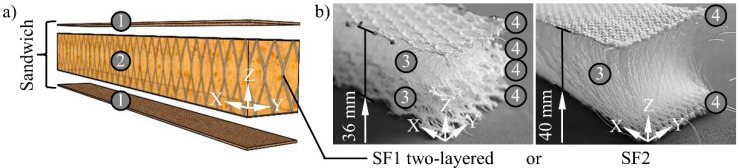
(**a**) Schematic explosion view of the investigated sandwich and (**b**) spacer fabrics used for reinforcement, 1—facing (thermoset reinforced with short glass fibres), 2—core (polyurethane foam reinforced with spacer fabric), 3—spacer threads, 4—warp-knitted fabric.

This paper deals with a sandwich consisting of short glass fibre reinforced thermoset facings and a PUR foam core which is continuously 3D reinforced with spacer fabric (Figure 1a). The investigations were carried out exemplarily with a total material thickness of 45 mm, which can be manufactured widely increased or reduced if required.

A vinyl ester (VE) resin of BÜFA Composite Systems GmbH & Co. KG (Rastede, Germany) was used for the manufacturing of the thermoset facings. It was cured with 1.5% ketone peroxide of the United Initiators GmbH (Pullach, Germany). Glass fibre mats with a fibre length of 50 mm from the Lange + Ritter GmbH (Gerlingen, Germany) named Rovicore were used to reinforce the facings. These have a thickness of 2.25 mm and a grammage of 900 g/m^2^. The calculated fibre volume content is 16% based on these values.

A 2-component cold foam system of the BASF Polyurethanes GmbH (Lemförde, Germany) was used for the manufacturing of the PUR. It generates a rigid foam with a high free foam weight per volume of 120 kg/m^3^ for high mechanical properties. It is intended in particular for use in the automotive industry. The system consists of an aromatic polyisocyanate and a polyol formulation containing all necessary additives for polyaddition and CO_2_ formation. The two components are processed in a ratio of 130 wt.% Iso to 100 wt.% Poly. The starting time of the system is 50 s and the tack-free time—2:30 min. An In-Mould-Coating (IMC) of the Fujichem Sonneborn Ltd. (London, UK) was used as release agent in the SRIM for the manufacturing of the PUR foam mouldings and as adhesion promoter between core and facings. It consists of a primer, curing agent and thinner which were processed in a ratio of 5:1:1. The setting time of the IMC was 3:30 min.

Spacer fabrics produced on HighDistance double-bar raschel machines of the Karl Mayer Holding GmbH & Co. KG (Obertshausen, Germany) are especially suitable for the reinforcement of large sandwich core thicknesses. The number of reinforcing layers and therefore the discontinuation of the fibre reinforcement in Z-direction can be minimised by their large thickness. Two corresponding variants were investigated in comparison to reinforce the core of the sandwich design (Figure 1b). These were produced by the Pressless GmbH (Flöha, Germany) and consist of polyethylene terephthalate (PET) fibres. The spacer threads have an IXI course and were worked of monofilaments. The fabric surfaces were produced as fillets from multifilaments. The variant SF2 was designed with the binding type of tricot. Additionally, fringe was used for SF1. Both variants differ in their structure by their thickness, yarn density and the resulting fibre volume content (Table 1).

A polyethylene (PE) foam mat was used in the manufacturing of the floor assembly as an additional sandwich core in areas with special acoustic insulation requirements. This was purchased from Potijk Bekleding & Acoustics BV (Almelo, The Netherlands).

### 2.2. Manufacturing Process

#### 2.2.1. Structural Reaction Injection Moulding (SRIM)

The manufacturing process was experienced by a beam geometry used for mechanical tests. A mould made of aluminium with three direct sprues and integrated electric heating was used to manufacture the cores in the SRIM. It was heated to 50 °C. The cavity was 500 mm long, 50 mm wide and 36 mm high. The IMC was sprayed again onto the cavity walls before each core was manufactured. Spacer fabrics were inserted to fabricate 3D continuous reinforced cores. These were cut into the area of the cavity of 500 mm × 50 mm with the USM-G electric circular blade of the Hoogs Cutting Systems GmbH & Co. KG (Bonn, Germany). The long side (X-direction in Figure 1) of the spacer fabrics corresponds to the working direction in the warp knitting process. Their thickness is compressed according to the cavity height when inserted into the mould. Two layers of SF1 were placed on top of each other to reinforce the cores. These did not have a textile connection. SF2 was used one-layered. The spacer fabrics have room temperature of about 23 °C when they are infiltrated by the foam because they are inserted shortly before the cavity is closed and the PUR reaction mass is introduced. A 2-component low-pressure machine from Unipre GmbH (Werl, Germany) was used to mix the polyol formulation with the polyisocyanate and for introducing the reaction mixture into the mould. An output of 600 g/min was used. Fixing of the spacer fabric against displacement was not necessary. The cavity is completely filled with PUR foam by the chemical reaction and the resulting blowing agent CO_2_. The tack-free time of the foam system defines the time until the samples can be demoulded. The IMC detaches from the mould surface and remains fully adhered to the cores. The resulting coating acts as an adhesion promoter to the facings in the further processing. For the complete filling of the cavity without inserts and minimal PUR reaction mass 165 g were required, which resulted in a foam weight per volume of 190 kg/m^3^. These values remained unchanged when SF1 two-layered was used as insert. The resulting core weight per volume was 235 kg/m^3^. The foam is compacting with SF2 as insert because of the high thread density in the cavity. A minimum of 210 g PUR reaction mass was required in this case which resulted in a foam weight per volume of 240 kg/m^3^ and a core weight per volume of 330 kg/m^3^.

#### 2.2.2. Vacuum Assisted Resin Transfer Moulding (VARTM)

The facings were manufactured on the cores using Vacuum Assisted Resin Transfer Moulding (VARTM). This is a special RTM process. It combines the applying of overpressure at the injection ports with vacuum at the vents. This allows higher pressure gradients within the cavity while avoiding bubbles through evacuation. The cores were treated with a spiked roller for the processing in VARTM. It had 8 mm long mandrels and was once pulled over the upper and lower foam surface. The diameter of the mandrels was 2.5 mm. Their distance was 20 mm in X-direction and 12 mm in Y-direction. The resin system can penetrate into the resulting openings in the core surfaces during the VARTM which increases the strength of the component transition area. The mould for the VARTM was made of Ureol. The cavity had a length of 1.6 m, a width of 0.5 m and a height of 45 mm. The height could be reduced to 4.5 mm to manufacture and characterise facing material separately. Thirty cores were positioned in the cavity with spacers between them. Two glass fibre mats each were placed above and below. The closed mould was exposed to a vacuum of 0.3 bar. The resin system was injected with a pressure pot also using 0.3 bar. About 11 kg were required to manufacture one panel. A resin trap was used for excess material and a demoulding time of 24 h for the post-curing of the resulting facings and the cooling of the sandwiches. The panels were separated into the beams of the SRIM again with a circular saw along the spacers after demoulding. The sandwich weight per volume without core reinforcement was 385 kg/m^3^. This is increased to 415 kg/m^3^ by SF1 two-layered and 500 kg/m^3^ by SF2. The density of the facings amounted to 1150 kg/m^3^.

#### 2.2.3. Upscaling

**Figure 2:**
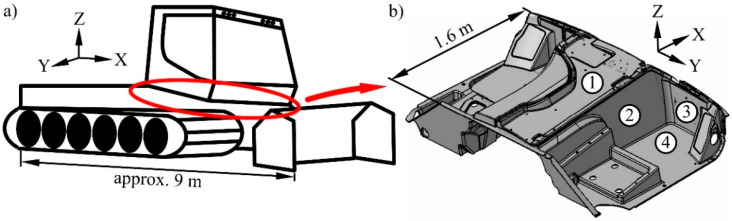
(**a**) Scheme of a snow groomer, (**b**) construction of the floor assembly, 1—sandwich with polyethylene (PE) foam core, 2—sandwich with polyurethane (PUR) foam core reinforced with polyethylene terephthalate (PET) spacer fabric, 3—sandwich with unreinforced PUR foam core, 4—plate without core, 1 to 4—short glass fibre-reinforced thermoset as facing.

The described process chain is upscaleable for producing large-volume applications with a complex shape. This was demonstrated for a floor assembly of a snow groomer with an area of 1.6 m × 2.2 m and a height of 0.8 m (Figure 2a). The construction was divided into four areas with different requirements. The composite material was used tailor-made to meet all objectives (Figure 2b). The design was carried out empirically because of the technological focus of the work on upscaling.

Each of the outer low-lying areas measuring 0.7 m × 1.6 m were designed alone in the short glass fibre-reinforced thermoset with a thickness of 4.5 mm. The FRP on its own is sufficient for the installation of the seats and the requirements of the footwell. Major mechanical requirements of the floor assembly resulted from the ROPS (Roll-Over Protective Structure) test of the snow groomer. The cabin strength is thereby checked during a single rollover. A defined survival space has to be maintained. The floor assembly received the highest mechanical load in this test on the side walls, which had an area of 0.8 m × 1.6 m. The sandwich design with fibre-reinforced PUR foam core in a thickness of 20 mm to 60 mm was used there for this reason. One to three layers of SF1 were used for the reinforcement. The side walls were connected through another sandwich section with an area of 0.8 m × 1.6 m and a thickness of 20 mm. Underneath this was the engine of the vehicle. The foam mat made of PE was used there as core for isolating the cabin acoustically. The frontal area of the floor assembly had an area of 0.8 m × 2.2 m and comes in contact with the environment. The sandwich design with a PUR foam core without reinforcement in a thickness of 20 mm was used there to ensure thermal insulation. The weight of the spacer fabric was saved there because of the lower mechanical requirements.

**Figure 3:**
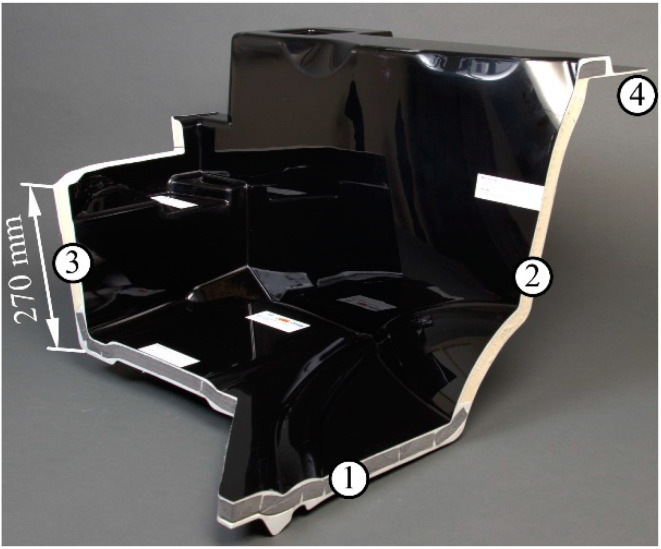
Segment of the floor assembly prototype in lightweight design, 1—sandwich with polyethylene (PE) foam core, 2—sandwich with polyurethane (PUR) foam core reinforced with polyethylene terephthalate (PET) spacer fabric, 3—sandwich with unreinforced PUR foam core, 4—plate without core, 1 to 4—short glass fibre-reinforced thermoset as facing.

The floor assembly was manufactured in principle according to the Section 2.2.1 and Section 2.2.2. Three moulds were developed for the production of the cores for the side walls and the front. About 1 kg of the 2-component cold foam system was processed in the RIM respectively SRIM for each part. Another mould with the component shape was developed for the VARTM. The core materials and the glass fibre mats were positioned in the cavity. About 45 kg of the VE resin system was necessary to infiltrate the remaining free space. The resulting component could be removed after the demoulding time (Figure 3).

The achieved mass of 55 kg demonstrates the great lightweight potential of the sandwich design. Conventional floor assemblies made of steel weigh up to 120 kg. One floor assembly without spacer fabrics in the side walls was produced as a reference. The reinforcement effect under application conditions could be investigated through a comparative mechanical component test in this way.

### 2.3. Mechanical Testing

#### 2.3.1. Quasi-Static

The testing machine Z100 and the contact extensometer multiXtens of the ZwickRoell GmbH & Co. KG (Ulm, Germany) were used for the quasi-static tests on samples. The facing material was characterised in the tensile test according to DIN EN ISO 527-4 (equivalent to ASTM D 3039) and the 3-point bending test according to DIN EN ISO 14125 (equivalent to ASTM D 7264). Standard-compliant samples were cut out of the 4.5 mm thick sheet material manufactured in the VARTM using a Computerised Numerical Control (CNC) waterjet cutting machine from the ATECH GmbH (Chemnitz, Germany).

The sandwiches were subjected to the quasi-static compression test according to DIN EN ISO 844 (equivalent to ASTM D 1621). The manufactured beams were cut into samples with the dimensions 50 mm × 50 mm × 45 mm for this purpose. The test stamp had a diameter of 78 mm and completely protruded the samples. The compression took place up to 30% residual height. The test speed was 4.5 mm per minute. Per series were 15 samples tested. A sample is destroyed according to the standard when the first force drop is recorded. The maximum force before this has to be used to calculate the compressive strength. The compressive stress at 10% compression should be used as characteristic value for comparisons if no force drop occurs during the whole test.

The quasi-static 3-point bending test of the sandwiches followed the DIN EN ISO 178 (equivalent to ASTM D 790). The beams were tested in the same dimensions as they were manufactured, which is 500 mm × 50 mm × 45 mm. Bending punch and support rolls had a radius of 5 mm. A span of 450 mm was used. The resulting ratio to the sample thickness is accordingly 10 which is one-third less than the value of 15 stated in the standard. The bending characteristic values are therefore not valid in the case of failure through delamination. Five samples per series were tested with a speed of 7.5 mm per minute. The test is finished according to the standard when the sample fractures or 5% strain is reached.

#### 2.3.2. Dynamic

**Figure 4:**
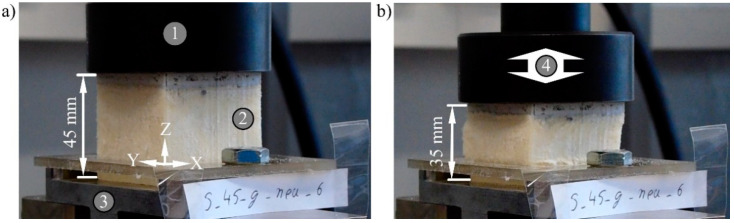
Sandwich in the fatigue compression test after (**a**) 50 cycles and (**b**) 100,000 cycles, 1—testing stamp, 2—sandwich sample, 3—base, 4—movement.

The fatigue tests were carried out with the servo-hydraulic testing machine 8501 of the Instron GmbH (Darmstadt, Germany), which determines the displacement by an incremental piston extensometer centrally above the test set-up. Sample size and stamp matched between fatigue and quasi-static compression testing (Figure 4). The loading of the samples was pulsating up to 0.40 MPa and down to 0.08 MPa. The samples were not completely unloaded to prevent resonance resulting from the impact of the test stamp.

Each sample was subjected to 10^6^ cycles at 10 Hz. This combination examines the fastest test speed that the inertia of the sandwiches allows while keeping a reasonable test duration of less than one week per sample for each load level investigated. The material-specific deformation is measured while the load peak is constant. A limit value was derived for the fatigue compression test using the results of the quasi-static test (Section 3.1). The samples are considered to be destroyed if this criterion is exceeded. The maximum load for a series was increased by 0.4 MPa when three samples of a sandwich type passed the fatigue test without failure. The load ratio (R) is accordingly in the range 1 < R < ∞.

**Figure 5:**
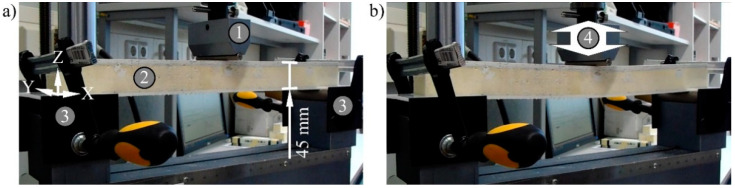
Sandwich in the fatigue bending test after (**a**) 50 cycles and (**b**) 100,000 cycles, 1—bending punch, 2—sandwich sample, 3—support roll, 4—movement.

The test setup and sample dimensions of the fatigue bending test were identical to the corresponding quasi-static procedure (Figure 5). Each beam was tested at a frequency of 1 Hz for 10^5^ cycles. The combination was chosen for the same reasons mentioned for the fatigue compression test. A constant peak displacement starting with 2 mm was set as the maximum load while the force was measured. The beams were fully unloaded in each cycle. This corresponds to a pulsating loading with R = −∞. The displacement was increased by 1 mm when three samples of a sandwich type had completed the test without fracture.

#### 2.3.3. Component Test

**Figure 6:**
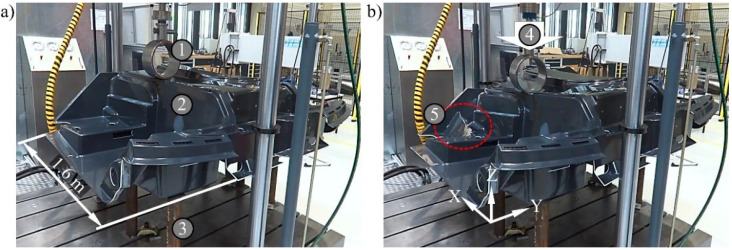
Floor assembly in the component test (**a**) at the beginning and (**b**) after 110 mm deflection, 1—test punch, 2—floor assembly, 3—support post, 4—movement, 5—failure location.

The floor assembly was mechanically characterised in a component test. The Z100 structure test stand from ZwickRoell GmbH & Co. KG (Ulm, Germany) was used for this. The supports were positioned at the points where the crawler track of the snow groomer would be attached. Six posts with a diameter of 80 mm were used for this at a distance of 1.3 m in Y-direction (Figure 6). The main load in the ROPS test is acting on the side walls. A corresponding point for the load application was chosen in the mechanical test carried out. The right-side wall was loaded centrally with a 250 mm diameter test punch. An incremental piston extensometer determines the deflection centrally over it. A maximum way of 110 mm and a test speed of 7.5 mm per minute were used. The force was measured. One floor assembly with and without spacer fabrics in the side walls was destroyed. The reinforcement effect can be assessed by comparing the measurement results.

## 3. Results and Discussion

### 3.1. Quasi-Static Mechanical Properties of the Facing Material and the Sandwiches

Table 2 shows the quasi-static tensile and bending properties of the facing material. The moduli are 20% and the strengths about 30% lower in Y- compared to X-direction. This anisotropy is caused by a slight preferential direction of the reinforcing glass fibre mat. The results correspond in general to normal mechanical properties of fibre-reinforced thermosets based on vinylester resin [37]. This indicates a defect-free manufacturing and correspondingly good material quality of the sandwich facings.

**Figure 7:**
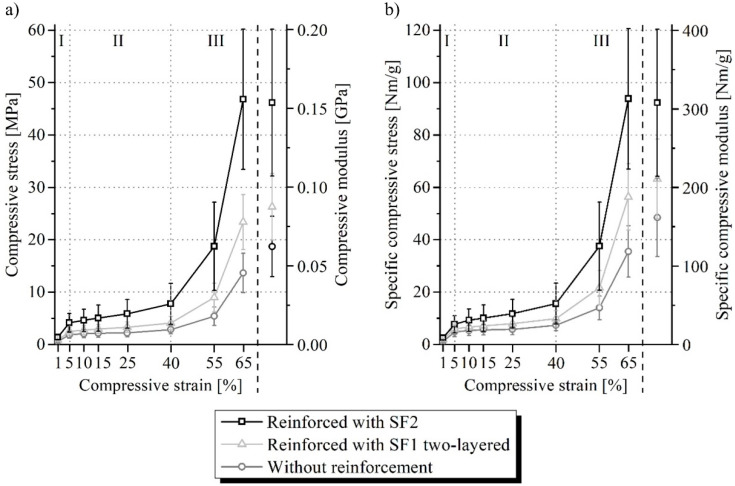
(**a**) Absolute and (**b**) specific quasi-static compression properties of the sandwiches, I—linear elasticity, II—plastic collapse, III—densification.

Mainly the core is loaded during the compression test of the sandwiches. The three deformation stages of PUR foams according to the state of the art were therefore demonstrated (Figure 7). The principal course is not changed by the fibre reinforcement with spacer fabrics. Cell walls and fibres bend elastically in the linear range up to about 5% compression. The plateau begins afterwards and continues up to about 40% compression. The cells collapse with or without reinforcement resulting in buckling and rupturing of the cell walls and fibres. These processes are irreversible. Material failure begins accordingly at the transition from the elastic to the plastic deformation.

A limit value of 10% compression was derived for the fatigue test based on these results. This is clearly shifted from the transition to the plastic deformation range in order to surely expect material failure. The third stage begins after 40% compression. The sandwiches are compressed thereby into compact blocks, which causes the steep curve rises.

No measurement curve showed a force drop in the quasi-static compression test. The results are therefore compared using stresses at defined strains (Figure 7a). The stress at 10% compression can be increased by 35% with SF1 two-layered and by 130% with SF2 compared to the sandwich with unreinforced core. Similar increases could be proven for the compressive modulus. The differences are even greater in the higher deformation range. The compressive stress at 65% deformation is increased by 70% with SF1 two-layered and by 240% with SF2. This is caused by the greater material compaction of the reinforced types with a higher weight per volume. Specific mechanical values are suitable for assessing the lightweight design potential of the sandwiches because the spacer fabrics bring additional weight into the composite and also compact the foam. Significant increases in the performance through the core reinforcement can nevertheless be demonstrated in this comparison (Figure 7b). These are 25% for SF1 two-layered and 80% for SF2 for the specific stress at 10% compression. Comparability is found for the increases in the specific compressive modulus. The specific compressive stress at 65% deformation was increased by 60% with SF1 two-layered and by 165% with SF2. A considerable reinforcing effect has accordingly been demonstrated. The PUR foam supports the stiffer fibres, which absorb the load and have to buckle before the cells are critically stressed. The one-layer reinforcement has achieved the higher characteristic values. This is because of the continuous reinforcement over the complete core thickness on the one hand. On the other hand, this spacer fabric structure also has a greater yarn density.

**Figure 8:**
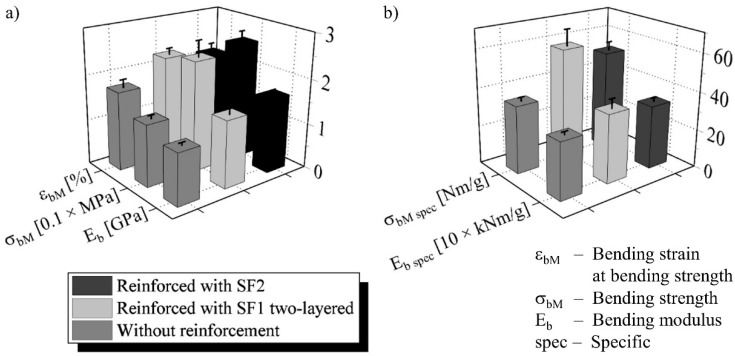
(**a**) Absolute and (**b**) specific quasi-static bending properties of the sandwiches.

No sample failed because of delamination in the quasi-static bending test. All characteristic values are therefore valid despite the reduced span. The foam without core reinforcement was plastically compressed after the facing above broke at the load application point. The core-reinforced sandwiches fractured brittle through the lower facing and the whole core with a slight curvature according to the spacer thread course. The bending modulus was increased by 30% with SF1 two-layered and by 45% with SF2 (Figure 8a). The improvement in bending strength was 80% or 90% and in the related bending strain—30% or 20%.

The highest specific bending characteristics were achieved by the sandwich with SF1 two-layered, which is in contrast to the absolute comparison (Figure 8b). The reason for this is the lower mass of the core with this spacer fabric, which is compensating for the higher absolute values of the type with SF2. Increases in specific bending modulus are 20% with SF1 two-layered and 10% with SF2. The specific strength is improved by 65% with SF1 two-layered and by 50% with SF2.

### 3.2. Fatigue Properties of the Sandwiches

**Figure 9:**
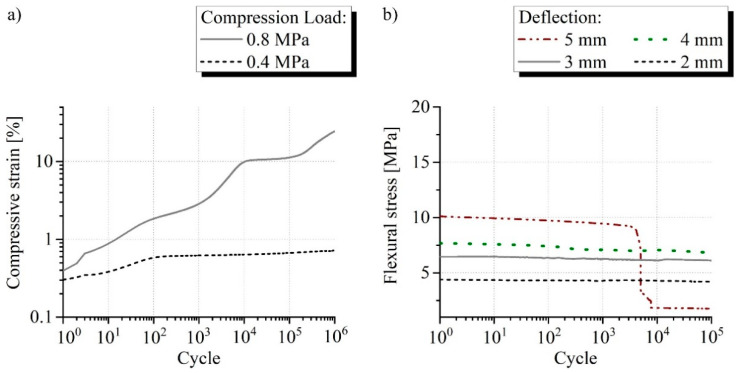
Typical measurement curves of the fatigue (**a**) compression and (**b**) bending test from the sandwich without core reinforcement comparing the investigated loads and displacements.

**Figure 10:**
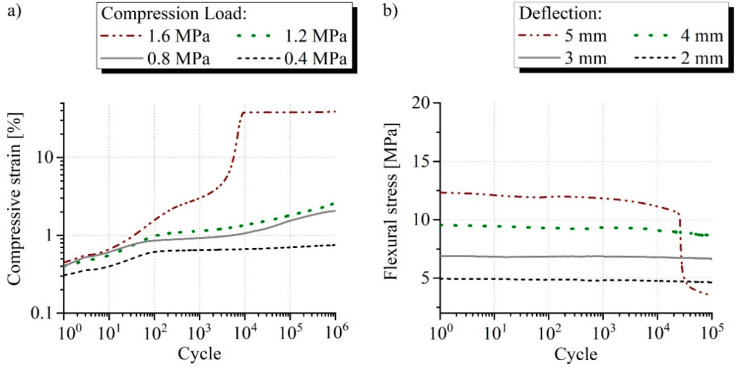
Typical measurement curves of the fatigue (**a**) compression and (**b**) bending test from the sandwich reinforced with SF1 two-layered comparing the investigated loads and displacements.

**Figure 11:**
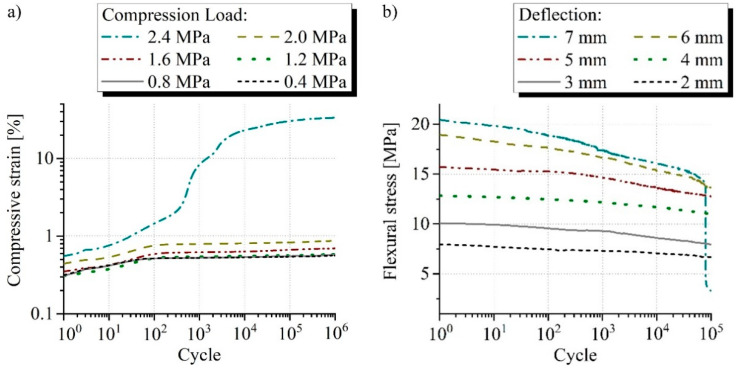
Typical measurement curves of the fatigue (**a**) compression and (**b**) bending test from the sandwich reinforced with SF2 comparing the investigated loads and displacements.

The results of the fatigue compression and bending test for the three sandwich types are presented in the Figure 9, Figure 10 and Figure 11. One exemplary curve is shown for each load investigated. No failure was found for the sandwich without core reinforcement in the fatigue compression test with 0.4 MPa, because the strain after 10^6^ cycles is far below 1% (Figure 9a). A compression of more than 10% and therefore plastic deformation of the material occurred after about 10^4^ cycles when the load was increased to 0.8 MPa. Fatigue strength for the sandwich without core reinforcement could only be proven for 0.4 MPa as a result. This corresponds to 15% of the quasi-static strength. The sandwich without core reinforcement failed under bending load at a deflection of 5 mm between 10^3^ and 10^4^ cycles (Figure 9b). The three load levels before were completed without defects. Long-term performance could accordingly be demonstrated at 4 mm, which corresponds to 0.5% bending strain. This is 30% of the result from the quasi-static test.

The sandwich with core reinforced by SF1 two-layered failed at a compression load of 1.6 MPa between 10^3^ and 10^4^ cycles (Figure 10a). Fatigue strength was therefore proven at 1.2 MPa, which corresponds to 45% of the quasi-static strength. Failure occurred also for this type of sandwich at a deflection of 5 mm when subjected to bending load (Figure 10b). This nevertheless took place later between 10^4^ and 10^5^ cycles and the stresses at the load levels are greater because of the core reinforcement. The long-term applicable bending strain of 0.5% is nevertheless only 20% of the corresponding quasi-static value.

The highest fatigue characteristics were found for the sandwich with core reinforcement by SF2. Failure in compression loading occurred at 2.4 MPa after about 10^3^ cycles (Figure 11a). The resulting fatigue strength of 2.0 MPa corresponds to 45% of the quasi-static strength like in the case of reinforcement with the other spacer fabric. The sandwich type with SF2 failed always shortly before reaching the 10^5^ cycles at 7 mm under bending load (Figure 11b). The deflection of 6 mm was permanently resisted and corresponds to 0.8% bending strain. This represents 40% of the quasi-static characteristic value.

The core reinforcement with spacer fabrics leads to clearly increased quasi-static mechanical properties according to the results presented in Section 3.1. These are also retained to a similar or much higher percentage under fatigue loading compared to the sandwich without core reinforcement. This is based mainly on the type of fatigue failure.

**Figure 12:**
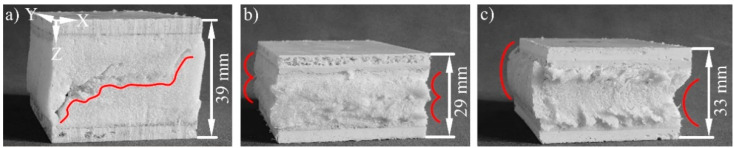
Failure modes of the sandwiches in the fatigue compression test: (**a**) diagonal crack through the core without reinforcement and (**b**) as well as (**c**) more than 10% plastic deformation of the cores with reinforcement corresponding to the curvature of the spacer threads, red lines—courses of the fractures.

A diagonal crack is formed through the sample with unreinforced core under compression load (Figure 12a). No macroscopic cracks were found in the reinforced cores. These are stopped by the fibres at their beginning. Only plastic deformation occurs as type of failure in these sandwiches (Figure 12b,c). This is oriented with the curvature of the spacer threads. The damage tolerance is therefore increased by the core reinforcement.

**Figure 13:**
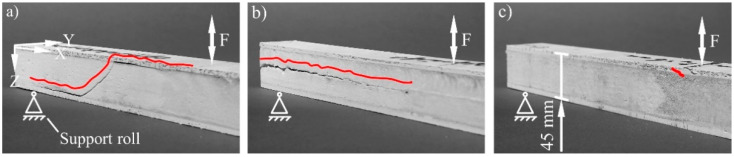
Failure modes of the sandwiches in the fatigue bending test: (**a**) diagonal crack through the core without reinforcement, (**b**) crack between the SF1 layers and (**c**) crack through the upper facing when reinforcing with SF2, red lines—courses of the fractures.

The unreinforced core is also destroyed by a diagonal crack under fatigue bending load, which extends along the facings (Figure 13a). Shear forces in the core and component transition area are the reason. This is in agreement with the results of previous work according to the state of the art. A horizontal crack develops when reinforcing with SF1 two-layered. (Figure 13b). This extends along the neutral fibre, where no stress is present. The weak point of this sandwich is this area, nevertheless. The two layers of the spacer fabric meet there without a textile connection (Figure 1b). They are joined in the SRIM by a thin and unreinforced PUR foam layer. A shear force-initiated crack in the core is dissipated through the fibres to this weak area, where it can expand freely. No core failure occurs with the single-layer reinforcement of SF2 (Figure 13c). The highest mechanical core properties without a weak point are achieved through the continuous fibre reinforcement in the Z-direction. The upper sandwich facing fails therefore vertically at the load application point because of compressive forces. A targeted crack dissipation or crack prevention can be achieved by equipping PUR foam cores with spacer fabrics. The damage tolerance under fatigue bending load is accordingly also improved by the reinforcement.

### 3.3. Mechanical Load Capacity of the Component

**Figure 14:**
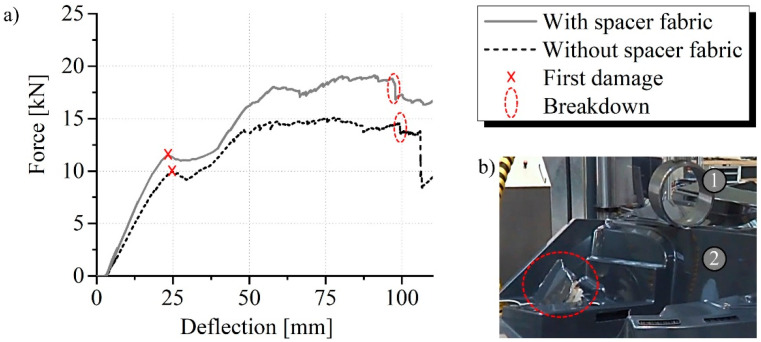
(**a**) Force measurement curves of the mechanical component testing and (**b**) detailed image of the breakdown, 1—test punch (D = 250 mm), 2—floor assembly.

The floor assembly failed in the mechanical component test with and without spacer fabric outside the sandwich area at the nearest post to the load application. Initial damage occurred there at about 25 mm deflection and progressed to breakdown at about 100 mm deflection (Figure 14b). A significantly increased stiffness of the floor assembly caused by the spacer fabrics is proven by the steeper rise of the force curve (Figure 14a). The core reinforcement of the side walls also improved the breaking force by 15% from 10.0 kN to 11.5 kN. The maximum force was increased by about 30% from 15 kN to nearly 20 kN. The reason for this is the absorption of the forces close to the introduction by the fibres of the spacer fabric and the subsequent distribution into the entire component.

## 4. Conclusions

This paper presents a thermoset sandwich design consisting of two short glass fibre reinforced facings and a foam in between. Spacer fabrics were investigated for reinforcing the core. Two variants with a different textile structure served for this purpose. A sandwich without core reinforcement was used as reference. It became clear that spacer fabrics take a dominating role for the mechanical performance of the sandwich design. Increased characteristic values were proven by the core reinforcement in a quasi-static compression and bending test. One example of this is the compressive stress at 10% deformation, which has been increased up to 130%. The bending strength was furthermore increased up to 90% and the related bending strain up to 30%. There are two reasons for this. The fibres of the spacer fabrics direct the forces through the composite structure according to the requirements on the one hand. The weight per volume of the PUR foam is increased by the use of spacer fabrics in the SRIM on the other hand. The differences in performance become therefore relative when the mass is taken into account. Increased specific characteristic values could nevertheless be demonstrated. The specific compressive stress at 10% deformation was increased by up to 80% and the specific bending strength by up to 65%.

The fatigue of the sandwich design was also investigated using the three variants and the two types of mechanical loading. Without core reinforcement retained 15% of the compressive stress at 10% deformation and 30% of the bending strain at bending strength when subjected to cyclic loading. The spacer fabrics caused a smaller decrease in both characteristic values. The maintenance with core reinforcement was up to 45% for the quasi-static compressive stress at 10% deformation and up to 40% for the bending strain at bending strength. Main reason for the improved fatigue is the changed failure mode. A diagonal crack through the foam always occurred without core reinforcement. The fibre reinforcement in the foam dissipated or prevented cracks. The damage tolerance of the sandwiches is accordingly improved by spacer fabrics. The results provide mechanical benchmarks for the design of the sandwich for applications subjected to fatigue.

The upscalability of the process chain consisting of SRIM and VARTM to large-volume components with complex geometry was also demonstrated. A floor assembly of a snow groomer was made completely of FRP with large areas in sandwich design. A weight saving of 55% was achieved compared to conventional floor assemblies made of steel. The new solution was characterised in a mechanical component test. It was also possible to investigate the reinforcing effect of spacer fabrics in this case of application based on a reference. An increase of the breaking force by 15% was proven.

## Figures and Tables

**Table 1:** Structural characteristics of the spacer fabrics (SF) investigated.

Designation	SF1	SF2
Thickness [mm]	18	40
Weight per volume [kg/m^3^]	32	62
Surface porosity [%]	65	8
Stitch density [1/in^2^]	70	260
Spacer thread density [1/in^2^]	140	520
Fibre volume content [%]	2.6	4.4
Diameter of monofilament [mm]	0.25	0.20

**Table 2:** Mechanical properties of the thermoset composite used as facing material.

*n* = 15	Tensile Properties		Bending Properties	
DIN-Norm	DIN EN ISO 527-4		DIN EN ISO 14125	
Tested direction	X	Y	X	Y
Young’s modulus [GPa]	5.6 ± 0.4	4.5 ± 0.4	7.2 ± 1.2	5.9 ± 0.9
Strength [MPa]	53 ± 8	35 ± 6	147 ± 44	111 ± 21
Breaking elongation [%]	1.5 ± 0.3	1.5 ± 0.4	2.4 ± 0.7	3.0 ± 0.5

Directions according to Figure 1; *n*—tested samples per method.

## Data Availability

The data presented in this investigation are available in the article.
